# Enzymatically Modified Starch Ameliorates Postprandial Serum Triglycerides and Lipid Metabolome in Growing Pigs

**DOI:** 10.1371/journal.pone.0130553

**Published:** 2015-06-15

**Authors:** Barbara U. Metzler-Zebeli, Eva Eberspächer, Dietmar Grüll, Lidia Kowalczyk, Timea Molnar, Qendrim Zebeli

**Affiliations:** 1 University Clinic for Swine, Department for Farm Animals and Veterinary Public Health, University of Veterinary Medicine Vienna, Vienna, Austria; 2 Anaesthesiology and Perioperative Intensive Care, Department for Companion Animals and Horses, University of Veterinary Medicine Vienna, Vienna, Austria; 3 Agrana Research & Innovation Center GmbH, Tulln, Austria; 4 Institute of Animal Nutrition and Functional Plant Compounds, Department for Farm Animals and Veterinary Public Health University of Veterinary Medicine Vienna, Vienna, Austria; National Institute of Agronomic Research, FRANCE

## Abstract

Developing host digestion-resistant starches to promote human health is of great research interest. Chemically modified starches (CMS) are widely used in processed foods and although the modification of the starch molecule allows specific reduction in digestibility, the metabolic effects of CMS have been less well described. This short-term study evaluated the impact of enzymatically modified starch (EMS) on fasting and postprandial profiles of blood glucose, insulin and lipids, and serum metabolome in growing pigs. Eight jugular-vein catheterized pigs (initial body weight, 37.4 kg; 4 months of age) were fed 2 diets containing 72% purified starch (EMS or waxy corn starch (control)) in a cross-over design for 7 days. On day 8, an 8-hour meal tolerance test (MTT) was performed with serial blood samplings. Besides biochemical analysis, serum was analysed for 201 metabolites through targeted mass spectrometry-based metabolomic approaches. Pigs fed the EMS diet showed increased (*P*<0.05) immediate serum insulin and plasma glucose response compared to pigs fed the control diet; however, area-under-the-curves for insulin and glucose were not different among diets. Results from MTT indicated reduced postprandial serum triglycerides with EMS versus control diet (*P*<0.05). Likewise, serum metabolome profiling identified characteristic changes in glycerophospholipid, lysophospholipids, sphingomyelins and amino acid metabolome profiles with EMS diet compared to control diet. Results showed rapid adaptations of blood metabolites to dietary starch shifts within 7 days. In conclusion, EMS ingestion showed potential to attenuate postprandial raise in serum lipids and suggested constant alteration in the synthesis or breakdown of sphingolipids and phospholipids which might be a health benefit of EMS consumption. Because serum insulin was not lowered, more research is warranted to reveal possible underlying mechanisms behind the observed changes in the profile of serum lipid metabolome in response to EMS consumption.

## Introduction

Resistant starch (RS) is a type of dietary fiber that includes all starch and starch degradation products being indigestible to mammalian enzymes in the small intestine [1]. Consumption of RS has been proven beneficial in treatment and prevention of lifestyle diseases, such as type-2-diabetes, dyslipidemia and obesity [2]. This has led dieticians to propagate the consumption of food ingredients naturally high in RS. Benefits were mostly related to the properties of RS to lower the acute blood glucose response and thus glycemic index of foods [3–5]. Evidence is growing that RS can lead to fundamental alterations in fat metabolism of hepatocytes and adipocytes, being reflected in elevated fat oxidation rates [6,7]. However, inconsistent reports exist regarding the potential of RS to reduce blood cholesterol and triglycerides [7,8] which can be partly associated with the great variation in physiological effects between the different types of RS.

Currently, RS is divided into 4 categories depending on the feature rendering RS indigestible for mammalian enzymes [2,9–11]. Three types of RS occur naturally in foodstuffs (RS1: physically inaccessible starch, RS2: native granular starch consisting of ungelatinized granules, RS3: retrograded amylose), whereas type 4 (RS4) cannot be found in nature and represents starch being chemically modified by esterification, crosslinking or transglycosylation [9,10]. Chemically modified starches (CMS) have become popular in nutrition and food science research recently as the chemical modification of the starch molecule allows reducing the digestibility of starch to a defined extent [2]. By far, CMS are commonly used in processed foods to improve rheological characteristics and texture of food items; and their consumption increases as the intake of processed foods expands [6,10,11]. The contribution of CMS to daily RS intake and their underlying protective physiological effects have been, however, less well explored than those of naturally occurring RS [2,11]. In considering that the chemical modification of the starch molecule is very specific, effects from one RS4 cannot be extrapolated to another CMS and potential health benefits need to be evaluated separately [2,11].

With blood metabolomics allowing quantification and identification of a very broad range of metabolites in a given biological system at a specific time point [12], a much deeper understanding of underlying biochemical processes in relation to dietary intervention can be gained than by studying single metabolites [13,14]. However, this powerful systems biological tool has not been largely used to explore metabolic effects of RS, in particular of potential RS4 candidates, in the pre- and postprandial state. Blood lipid metabolites, such as lysophospholipids and sphingolipids, for instance, are important extracellular signaling molecules and have been recognized as candidate biomarkers for early diagnosis of lifestyle diseases such as insulin resistance [15,16]. The pig is an attractive model to study digestive-metabolic responses to nutritional intervention due to the superior similarity in digestive physiology, anatomy and metabolism compared to rodent models [11,17,18]. Moreover, implementation of permanent catheters in pigs allows repeated blood samplings [19]. This, and the fact that pigs readily eat the dietary test component at higher inclusion levels than humans would eat, helps standardizing the experimental settings and attributing physiological findings to the actual test component.

In applying a meal tolerance test, this study aimed at investigating the hypothesis whether enzymatic modification of starch improves the postprandial blood glucose, insulin and lipid response and modulates fasting and early postprandial serum metabolome compared to rapidly digestible waxy corn starch using jugular vein-catheterized growing pigs in a short-term experiment. In order to minimize confounding effects of other non-starch carbohydrates attached to starch molecules in plant feedstuffs, high levels of purified starches were used in the experimental diets.

## Materials and Methods

### Ethical statement

All procedures involving animal handling and treatment were approved by the institutional ethics committee of the University of Veterinary Medicine Vienna (Vienna, Austria) and the national authority according to §26 of the Law for Animal Experiments, Tierversuchsgesetz 2012—TVG (GZ 68.205/0062-II/3b/2013).

### Animals, housing and surgery

Eight crossbred castrated male growing pigs (initial body weight: 37.4 [S.D. 2.69] kg; 4 month of age; [Landrace × Large White] × Piétrain) were used in this study. One week before catheterization surgeries, pigs were moved into individual metabolism cages (1.0 × 1.2 m) in which they remained until the end of the study. The cages were made of Plexiglas walls and slatted flooring and were equipped with a heating lamp, single-space feeder and a nipple drinker. Demineralized water was continuously available. The room temperature was about 20 to 21°C, and it was checked several times daily to assure that pigs did not feel too hot or cold. As environmental enrichment, pigs received new empty plastic water bottles and surgical gauze as chewing material every day.

Pigs were surgically fitted with a 1-m-long polyethylene catheter (TYGON S-54-HL Medical Tubing; inner diameter 1.016 mm; outer diameter 1.778 mm; Saint-Gobin, Akron, OH, USA) in the jugular vein. An 18-gauge blunt needle (Sterican single use needle; B. Braun Melsungen AG, Melsungen, Germany) was used as adaptor and one 2-mm ring of tubing was installed 10 cm from the catheter tip glued with Loctite 406 (Loctite Corp., Rocky Hill, CT, USA). Edges were made smooth with a file after complete drying. The tips of the catheters were cut with an angle of 45° using a scalpel blade to enlarge the surface opening and the tip was further cut at an angle of 90° and smoothened with a file to prevent irritation of the blood vessel and blood coagulation. Pigs were sedated via an intramuscular injection of ketamine (7 mg/kg; Narketan, Vétoquinol AG, Ittigen, Austria) and midazolam (1 mg/kg; Midazolam ERWO, Erwo Pharma, Brunn am Gebirge, Austria). After placement of a lateral ear vein catheter and induction with propofol (titrated to effect; Propofol 1%, Fresenius, Graz, Austria), pigs were intubated, connected to an anesthetic machine and mechanically ventilated with 1–2.5% isoflurane (Vetflurane, Virbac, Vienna, Austria) in oxygen. Analgesia was further provided by a continuous rate infusion (CRI) of fentanyl (0.3 μg/kg/min; Fentanyl-Janssen, Janssen-Cilag, Vienna, Austria). Pigs obtained a CRI of lactated Ringer’s solution (10 ml/kg/h; Fresenius, Graz, Austria). Cardiovascular and respiratory parameters as well as body temperature were monitored throughout the surgical procedure and kept constant. Once fully recovered from anesthesia, pigs returned to their home cages. The catheter was tunneled dorso-caudally beneath the skin and exteriorized at the left side of the nape close to the spine. Catheters were secured using pouches made of 10 × 15 cm-wide strips of generic bandage and elastic adhesive bandage and were flushed aseptically daily with 5 ml of 25 IU of heparinized normal saline to maintain their patency. The day prior to catheterization surgery, pigs received analgesic treatment (Metacam; 0.4 mg/kg BW; meloxicam; Boehringer Ingelheim, Ingelheim, Germany) and on the day of surgery analgesic and antibiotic treatment (Cobactan; 2 mg/kg BW; cefquinon sulfate; Intervet GesmbH, Vienna, Austria). Postoperative management included before mentioned antibiotics and analgesics for 3 d. All efforts were made to minimize suffering. At the end of the study, pigs were anesthetized (Narketan, 10 ml/kg body weight; Ketamine HCl; Vétoquinol AG, Ittigen, Austria; and Stresnil, 3 ml/kg body weight; Azaperone; Biokema SA, Crissier, Switzerland) and euthanized by intracardiac injection of T61 (10 ml/kg; Embutramide; MSD Animal Health, Vienna, Austria).

### Diets

Two semi-purified experimental diets were prepared of 718 g/kg refined corn starch, 180 g/kg casein, 40 g/kg cellulose in the form of FibreCell M1 (agromed Austria GmbH, Kremsmünster, Austria), and rapeseed oil. Both diets were fortified with vitamins and minerals (Table 1) to meet current nutrient requirements [20]. Diets were formulated to be isocaloric on gross energy basis (Table 1). The control diet contained standard rapidly digestible waxy cornstarch (Agrana Stärke GmbH, Gmünd, Austria), whereas the test starch diet contained Agenanova (Agrana Stärke GmbH, Gmünd, Austria). Agenanova is an enzymatically modified cornstarch product (EMS) prepared from waxy cornstarch which consists in uniform molecular weight distribution and increased branching of the starch molecules. According to the manufacturer, the amylopectin fraction of the EMS contains twice as many α-1,6-glycosidic bonds (approximately 8%) in comparison to the non-modified native waxy cornstarch (4% α-1,6-glycosidic bonds). Because the enzymatic treatment of waxy cornstarch also reduced the molecular weight, the dextrose equivalent (DE), as a measure of the amount of reducing sugars relative to glucose (dry matter basis), of the EMS was determined which was below 1 indicating that almost no low-molecular sugars were present. Mono- and disaccharides were absent to the greatest possible extent.

**Table 1:** Ingredients and analyzed chemical composition of the experimental diets.

	Control diet	EMS diet
Ingredient (%)		
Waxy cornstarch	72.1	-
Enzymatically modified starch^a^	-	72.1
Casein	18.0	18.0
Lignocellulose^b^	4.0	4.0
Rapeseed oil	1.0	1.0
Vitamin-mineral-premix^c^	4.3	4.3
Monocalcium phosphate	0.6	0.6
Analyzed chemical composition (dry matter basis, g/kg)		
Dry matter	938	949
Crude protein	167	166
Total starch^d^	668	687
Resistant starch^e^	6.6	1.5
Calcium	8.1	8.0
Phosphorus	5.0	4.8
Gross energy (MJ/kg)	16.8	16.9

^a^Agenanova (AGRANA Stärke, Gmünd, Austria)

^b^FibreCell (agromed Austria GmbH, Kremsmünster, Austria)

^c^Provided per kilogram of complete diet (GARANT GmbH, Pöchlarn, Austria): 16,000 IU of vitamin A, 2,000 IU of vitamin D_3_, 125 mg of vitamin E, 2.0 mg of vitamin B_1_, 6.0 mg of vitamin B_2_, 3.0 mg of vitamin B_6_, 0.03 mg of vitamin B_12_, 3.0 mg of vitamin K_3_, 30 mg of niacin, 15.0 mg of pantothenic acid, 900 mg of choline chloride, 0.15 mg of biotin, 1.5 mg of folic acid, 200 mg of vitamin C; 4.6 g of Ca, 2.3 g as digestible P, 2.4 g as Na, 2.0 g of Cl, 3.2 g K, 1.0 g Mg; 50 mg of Mn (as MnO); 100 mg of Zn (as ZnSO_4_); 120 mg of Fe (as FeSO_4_), 15.6 mg of Cu (as CuSO_4_), 0.5 mg of Se (as Na_2_SeO_3_), 1.9 mg of I (as Ca(IO_3_)_2_), 3 g TiO_2_.

^d^Total starch was determined using the Total Starch Assay Kit (K-TSTA; Megazyme International Ireland Ltd., Bray, Ireland).

^e^Resistant starch was determined using the Resistant Starch Assay Kit (K-STAR; Megazyme International Ireland Ltd., Bray, Ireland).

Pre-surgery and during the 5-day recuperation period post-surgery, pigs were fed a commercial grower diet (1,100 g per day divided into two meals; ME, 13 MJ/kg, CP, 16.5%, as-fed basis). Pigs were not fed on the day of surgery. The next morning, pigs were fed 200 g and 400–500 g in the afternoon. On the second day after surgery, pigs were fed pre-surgery feed amounts.

Diets were analyzed in duplicate for dry matter, ash, total starch, protein, Ca and P using classical methods [21]. Total and resistant starch contents of diets were determined using commercial enzymatic kits (K-TSTA and K-STAR; Megazyme International Ireland Ltd., Bray, Ireland). Gross energy content of diets was measured by combustion using a bomb calorimeter (Calorimeter C200 System, IKA-Werke GmbH & Co KG, Staufen, Germany).

### Experimental design, measurements and sample collection

The study was designed as a crossover dietary intervention study. On day 6 after surgery, pigs were randomly assigned to one of the 2 experimental diets (control or EMS diet). In each of the 2 experimental periods, 4 pigs received the control diet and 4 pigs were allotted to the EMS diet; amounting in total to 8 genuine observations for the control diet and 8 genuine observations for the EMS diet. Each 8-d experimental period consisted of a 7-day acclimation to the diets, followed by an 8-hour meal tolerance test (MTT) the next day. The length of the acclimation to the diets was comparable to previous short-term studies on RS in growing pigs [5]. The MTT is a gold standard measure of endogenous insulin secretion in humans [22] and pigs [5], in which after an overnight fast a standardized carbohydrate-rich meal is consumed and blood samples are repeatedly taken [22].

The daily feed allowance was adjusted to approximately 3 × maintenance of energy (3 × 0.44 MJ ME/kg BW^0.75^)[20] and was fed as mash in two equal meals at 8:00 am and 4:00 pm. Control and EMS diets were mixed with water in a ratio of about 2: 1 (vol/vol). During the acclimation to the diets, pigs ate between 1032 and 1220 g of the control diet (dry matter basis) and between 1044 and 1234 g of the EMS diet (dry matter basis) per day. The meal size during the present MTT was similar to the feed amount offered to the pigs per meal on day 7 of the acclimation phase as it is critical to standardize the evening meal and portion size before studying glycemic index of foods [23]. Thus, during the MTT the amount of diet (before mixing with water) was 650 g for the control diet and EMS diet on fresh matter basis which corresponded to 610 g and 617 g dry matter for the control diet and EMS diet, respectively. The MTT was performed in 4 pigs per day, resulting in 2 consecutive days of MTT in each experimental period. With their last meal at 4:00 pm the previous day, pigs were fasted for 15 hours before sampling of baseline (fasting) blood samples. Serial blood samples were taken at -30 (fasting blood sample), 30, 45, 60, 90, 120, 150, 180, 210, 240, 300, 360, 420, and 480 min postprandially. The volume of the baseline blood sample was 20 mL and 12 mL for all postprandial blood samplings. After blood collection, fluid loss was replaced by the respective volume of sterile physiological saline (Fresenius Kabi Austria GmbH, Graz, Austria) and the catheter was flushed with 2 mL of 5 IU/mL heparinized saline to prevent clotting. Blood samples for serum metabolomics were taken preprandially (-30 min) and at 60 min postprandially. Blood for metabolomics, triglycerides, cholesterol, NEFA, insulin and haptoglobin was collected in serum tubes (S-Monovette 9.0ml Z; Sarstedt GmbH & Co, Nümbrecht, Germany) and for glucose and lactate in fluoride-EDTA tubes (S-Monovette 2.7ml FE; Sarstedt GmbH & Co). Serum and fluoride-EDTA tubes were stored immediately on ice before being centrifuged at 1,811×g for 10 min (Eppendorf Centrifuge 5810 R, Eppendorf, Hamburg, Germany). Plasma and serum aliquots were frozen at -80°C for metabolomics and insulin assays and at -20°C for glucose, lactate, triglycerides, cholesterol, NEFA and haptoglobin.

### Biochemical variables

Because RS repeatedly showed to influence blood glucose and insulin metabolic responses as well as to potentially affect postprandial blood lipid profiles, we decided to measure these sentinel plasma and serum blood parameters. Haptoglobin was selected to investigate whether the dietary starch type influenced reactants of the innate immune system. In this context, plasma glucose, plasma lactate, serum triglycerides, serum cholesterol and serum NEFA were determined by standard enzymatic colorimetric analysis using an autoanalyzer for clinical chemistry (Cobas 6000/c501; Roche Diagnostics GmbH, Vienna, Austria) for all MTT blood samplings. Porcine specific commercial ELISA kits were used to analyze serum concentration of insulin (Mercodia, Uppsala, Sweden) for all MTT blood samplings and fasting haptoglobin (Genway, San Diego, CA, USA) according to the manufacturer’s instructions.

### Metabolome profiling

In order to cover a broader spectrum of small-sized metabolites that belong or are interconnected to the sentinel blood parameters, metabolome profiling was performed during the fasting state and at 60 min postprandially, using a targeted metabolomics approach based on electrospray ionization liquid chromatography–mass spectrometry (ESI-LC-MS/MS). Measurements were conducted with the Absolute*IDQ* p180 kit and Energy Metabolism Assay of BIOCRATES Life Sciences AG (Innsbruck, Austria). The Absolute*IDQ* p180 kit allows a simultaneous quantification of 186 metabolites (free carnitine, acylcarnitines (Cx:y), amino acids (proteinogenic amino acids, citrulline and ornithine), biogenic amines, sum of hexoses, glycerophospholipids (lysophosphatidylcholines (lysoPC) and phosphatidylcholines (PC diacyl (aa) and acyl-alkyl (ae)), and sphingolipids (SMx:y)) out of 10 μL blood serum, whereas the Energy Metabolism Assay detects 15 metabolites out of 30 μL blood serum (3-phosphoglycerate, α-ketoglutaric acid, adenosine-3’, 5’-cyclic monophosphate, arginine, aspartate, dihydroxyacetonephosphate and 3-phosphoglyceraldehyde, fumarate, glutamate, hexose, hexosephosphate, lactate, pentosephosphate, pyruvate and oxaloaceate, succinate, tetrosephosphate). All analyses were performed by the Target*IDQ* Service of BIOCRATES Life Science AG. Both kits were fully automated assays and based on phenylisothiocyanate-derivatization in the presence of internal standards. Identification of targeted classes of metabolites was performed using mass spectrometry (4000 QTRAP system; Applied Biosystems/MDS Sciex, Foster City, CA, USA). Mass chromatograms were analyzed using BIOCRATES software (BIOCRATES Life Science AG) and internal standards served as reference to calculate metabolite concentrations and analytical variance and accuracy of measurements were determined as described recently [14].

### Calculations and statistical analysis

Because 1 and 2 catheters of pigs malfunctioned during measurement days of experimental period 1 and 2, respectively, 6 observations from pigs fed the EMS diet and 7 observations from pigs fed the control diet were available for calculation of area-under-the-curve (AUC) and statistical analysis of postprandial data. For the -30 min (fasting) blood sample, 7 observations for the EMS diet and 7 observations for the control diet were available.

Because of the sharp turning points of the curves of most of the target parameters (glucose, insulin, lactate and cholesterol), computation of AUC values was performed using the trapezoidal rule in SAS (version 9.3; SAS Inst. Inc., Cary, NC, USA). For plasma glucose and serum insulin, AUCs were computed from 0–480 min postprandially. For plasma lactate and serum lipids, AUCs from 0–480 min postprandially were calculated. The latter method is used to approximate the AUC by dividing the area into a number of strips of equal width of which the area of the trapezium formed is approximated. The sum of these approximations gives the final results of the AUC.

To compare differences among diets, data were subjected to ANOVA using the MIXED procedure of SAS (version 9.3; SAS Inst. Inc., Cary, NC, USA) using the pig as experimental unit. Means were reported as least-squares means ± standard error of the mean (SEM) and *P*≤0.05 and *P*<0.10 were defined as significance and trend, respectively. For serum haptoglobin, fixed effects of diet and experimental period as well as the random effect of pig within period were included in the main model. Data of MTT and serum metabolome data were analyzed as repeated measures over time including the fixed effects of experimental period, diet, time and diet×time. For serum metabolomics data, multiple comparisons between diet groups were performed separately for each sampling time (fasting state and 60 min postprandially) using the probability of difference. Degrees of freedom were approximated using Kenward-Rogers method (ddfm = kr).

## Results

### Meal tolerance test

**Fig 1:**
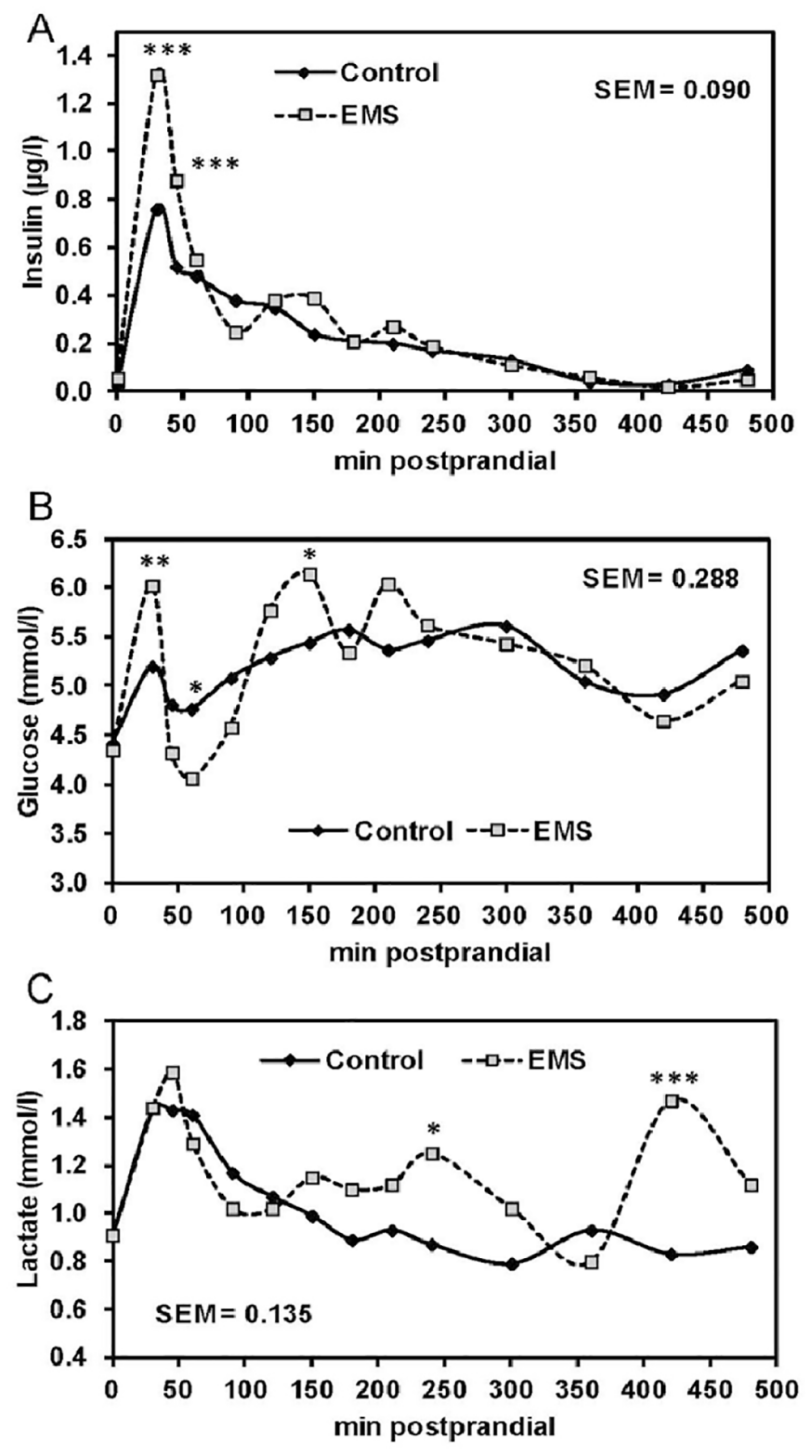
Serum concentrations of insulin (A), glucose (B), and lactate (C) during the meal tolerance test in pigs fed the enzymatically modified starch (EMS) diet or control diet. Results are presented as least-squares means ± SEM (control diet, n = 7; EMS diet, n = 6). EMS diet versus control diet, *P<0.10, **P<0.05, ***P<0.001.

**Fig 2:**
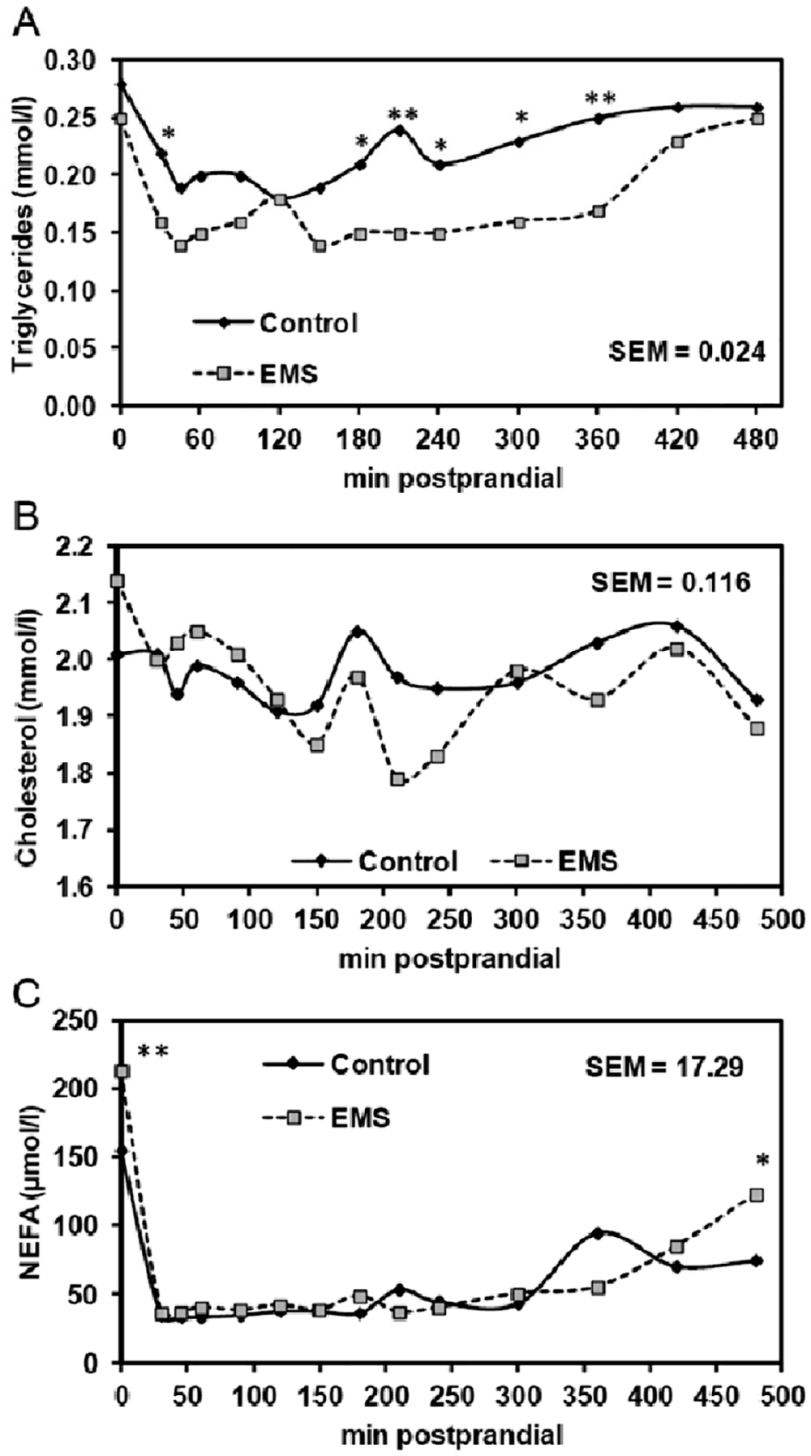
Serum concentrations of triglycerides (A), cholesterol (B), and non-esterified fatty acids (NEFA) (C) during the meal tolerance test in pigs fed the enzymatically modified starch (EMS) diet or control diet. Results are presented as least-squares means ± SEM (control diet, n = 7; EMS diet, n = 6). EMS diet versus control diet, *P<0.10, **P<0.05.

Replacing the standard waxy corn starch by EMS was well tolerated by pigs which consumed the entire amounts of 617 and 609 g of EMS diet and control diet offered per meal (dry matter basis) on MTT days within 25 min. Pigs fed the EMS diet had similar fasting concentrations of glucose, insulin, lactate (Fig 1A–1C), triglycerides, and cholesterol (Fig 2A and 2B) compared to pigs fed the control diet, whereas the concentration of NEFA was raised (*P* = 0.016) by 37% in pigs fed the EMS diet compared to those fed the control diet (Fig 2C). Serum insulin peaked at 30 min postprandially and pigs fed the EMS diet had a 74%-greater insulin concentration at 30 min compared to those fed the control diet (Fig 1A). At 45 min postprandially, serum insulin concentration decreased but remained elevated (*P* = 0.006) in pigs fed the EMS diet compared to those fed the control diet. Afterwards, serum insulin concentrations similarly decreased over time for both diets until reaching fasting state concentrations at 360 min postprandially. Because we sampled a peripheral blood vessel, there was no clear plasma glucose peak for the control diet with plasma glucose concentrations in the immediate postprandial phase (Fig 1B). However, the course of plasma glucose highly fluctuated in pigs fed the EMS diet compared to pigs fed the control diet. Pigs fed the EMS diet showed three glucose peaks at 30, 150 and 210 min postprandially, being higher at 30 (*P*<0.05) and 150 min (*P*<0.10) compared to pigs fed the control diet. Additionally, pigs fed the EMS diet had plasma glucose concentrations between 45 and 90 min postprandially which were below those of pigs fed the control diet and tended (*P*<0.10) to be reduced at 60 min postprandially. Postprandial plasma glucose concentrations did not reach the preprandial concentration again during the 8-hour MTT. The course of postprandial lactate (Fig 1C) was similar to serum insulin in pigs fed the control diet with a peak at 30 to 60 min postprandially and decreasing thereafter to preprandial concentrations until 180 min. In contrast, the course of plasma lactate in pigs fed the EMS diet showed three peaks at 45, 240 and 420 min postprandially, with lactate concentrations at 240 (*P*<0.10) and 420 min (*P*<0.01) being higher than those in pigs fed the control diet. Serum triglycerides were lower (*P*<0.05) at 60 min after feeding compared to the fasting triglyceride concentration, thereby pigs fed the EMS diet had an even lower triglyceride concentration than pigs the control diet (Fig 2A). Triglyceride concentrations increased in serum starting from 150 min postprandially in pigs fed the control diet, whereas in pigs fed the EMS diet serum triglycerides were increased starting from 360 min postprandially and reached the preprandial concentration at 480 min. Overall, pigs fed the EMS diet had lower (*P*<0.05) serum triglyceride concentrations at 180 and 360 min and tended (*P*<0.10) to have lower concentrations at 30, 210, 240 and 300 min postprandially. Serum NEFA concentrations substantially decreased after feed consumption (Fig 2C) and were similar for both diets until 480 min postprandially when pigs fed the EMS diet tended (*P*<0.10) to have higher concentrations compared to pigs fed the control diet.

There was no significant diet effect on AUC 0–480 min values for glucose, insulin, cholesterol and NEFA (Table 2). The AUC 0–480 min of lactate and triglycerides, however, tended (*P*<0.10) to be higher in pigs fed the EMS diet compared to those fed the control diet. No difference in serum haptoglobin concentrations was found in pigs fed the control diet compared to pigs fed the EMS diet (1.12 versus 1.25 ± 0.272 mg/ml for pigs fed control and EMS diet, respectively).

**Table 2:** Area-under-the-curve 0–480 min values during the meal tolerance test in pigs fed the enzymatically modified starch (EMS) diet or control diet.

	Control diet	EMS diet	SEM^a^	*P*-value
Glucose (mol/l × 480 min)	2.5	2.5	0.06	0.97
Insulin (μg/l × 480 min)	100	116	13.5	0.27
Lactate (mmol/l × 480 min)	471	560	34.4	0.08
Triglycerides (mmol/l × 480 min)	108	84	8.4	0.06
Cholesterol (mmol/l × 480 min)	953	927	50.1	0.63
NEFA (mmol/l × 480 min)^b^	27	28	3.6	0.75

^a^Values are least squares means ± SEM; control diet, n = 7; EMS diet, n = 6.

^b^NEFA, non-esterified fatty acids.

### Serum metabolome profiles

The MS/MS targeted analysis provided results for 124 metabolites (Tables 3 and 4; S1 and S2 Tables). Besides 8 out of 15 intermediates of energy metabolism, the sum of hexoses (including glucose), 35 amino acids and biogenic amines, 4 acylcarnitines, 13 sphingolipids, and 79 phospholipids including lysophosphatidylphosphates and phospholipids were detected using the Absolute*IDQ* p180 kit.

**Table 3:** Selected serum metabolite concentrations (μmol/l) of pigs fed the enzymatically modified starch (EMS) diet or control diet in the fasting state and at 60 min postprandially.

	Fasting state			60 min postprandially			*P*-value		
Metabolite (μmol/l)^a^ ^–^ ^d^	Control	EMS	SEM	Control	EMS	SEM	Diet	Time	Diet × Time
Intermediates of energy metabolism									
Sum of hexoses	5831	5494	259.6	6228^a^	5144^b^	269.4	0.06	0.94	0.26
Lactate	500	475	62.0	764^a^	707^b^	63.6	0.39	0.001	0.72
α-Ketoglutaric acid	10.2	10.4	1.00	12.1^b^	13.6^a^	1.03	0.38	0.025	0.48
Fumarate	4.0	4.1	0.31	4.8	5.5	0.33	0.24	0.011	0.46
Pyruvate+oxaloacetate	74.0	71.8	5.99	83.3	84.6	6.16	0.92	0.045	0.71
Acylcarnitines									
C0	3.3	3.1	0.11	3.6	3.4	0.11	0.14	0.026	0.95
C2	0.29	0.27	0.01	0.34	0.34	0.01	0.38	0.005	0.70
C14:1	0.023	0.025	0.001	0.02	0.02	0.002	0.68	0.971	0.47
C16	0.024	0.025	0.002	0.033^b^	0.038^a^	0.002	0.16	<0.001	0.31
Lysophosphatidylcholines									
lysoPC a C16:1	2.0^b^	3.8^a^	0.31	1.8^b^	4.0^a^	0.32	<0.001	0.89	0.36
lysoPC a C17:0	0.60^b^	0.72^a^	0.04	0.59	0.71	0.04	0.012	0.78	0.98
lysoPC a C18:1	21.8^b^	30.1^a^	1.67	19.3	31.0	1.77	<0.001	0.54	0.20
lysoPC a C18:2	5.5A	4.7B	0.30	5.7^a^	4.6^b^	0.31	0.015	0.75	0.52
lysoPC a C20:3	2.1^b^	2.6^a^	0.13	2.1	2.6	0.15	0.005	0.36	0.68
Sphingomyelins									
SM(OH) C22:1	3.0	2.7	0.18	2.9	2.5	0.19	0.098	0.36	0.75
SM(OH) C24:1	0.38A	0.29B	0.03	0.38^a^	0.26^b^	0.03	0.015	0.62	0.59
SM C18:0	14.8A	12.9B	0.97	14.5	12.7	0.99	0.032	0.68	0.98
SM C18:1	4.2	3.9	0.25	4.3	3.9	0.26	0.083	0.90	0.83
SM C24:0	12.3	10.9	0.75	12.5	10.1	0.77	0.031	0.63	0.48
SM C24:1	27.8A	24.0B	1.52	27.5	22.6	1.57	0.020	0.52	0.67
SM C26:1	0.11^a^	0.03^b^	0.02	0.06	0.04	0.02	0.053	0.47	0.24
Phosphatidylcholines									
PC aa C30:0	1.5^b^	1.8^a^	0.12	1.4^b^	1.7^a^	0.12	0.010	0.31	0.89
PC aa C32:1	10.6	19.5	1.66	8.3	18.1	1.70	<0.001	0.12	0.68
PC aa C32:2	0.30^b^	0.72^a^	0.10	0.14	0.67	0.10	0.001	0.19	0.50
PC aa C34:1	218.8^b^	269.3^a^	13.59	200.1^b^	255.3^a^	14.01	0.005	0.18	0.83
PC aa C34:2	71.3	63.0	4.01	70.2^a^	58.2^b^	4.12	0.021	0.36	0.57
PC aa C34:3	3.4^b^	4.2^a^	0.31	3.0^b^	4.3^a^	0.32	0.009	0.54	0.42
PC aa C34:4	0.27^b^	0.36^a^	0.03	0.22^b^	0.35^a^	0.03	0.007	0.29	0.53
PC aa C36:1	126.5^b^	165.5^a^	9.68	110.9^b^	160.9^a^	9.99	0.003	0.25	0.52
PC aa C36:5	4.1B	5.6A	0.50	3.3^b^	5.7^a^	0.52	0.008	0.46	0.35
PC aa C36:6	0.15	0.19	0.02	0.11^b^	0.21^a^	0.02	0.011	0.75	0.18
PC aa C38:4	128.6A	108.3B	7.87	121.5A	101.9B	8.13	0.028	0.33	0.96
PC aa C38:6	20.5A	17.1B	1.36	18.5	16.1	1.39	0.029	0.17	0.74
PC aa C40:3	1.5	1.3	0.12	1.5	1.8	0.12	0.044	0.76	0.31
PC aa C40:5	22.5	19.9	1.38	21.1	18.5	1.43	0.09	0.28	0.93
PC aa C40:6	18.8	16.1	1.13	16.4	14.9	1.17	0.08	0.11	0.57
PC aa C42:1	0.11	0.10	0.01	0.12	0.12	0.01	0.99	0.049	0.24
PC aa C42:2	0.15	0.13	0.01	0.15	0.13	0.01	0.05	0.83	0.68
PC aa C42:4	0.18A	0.15B	0.01	0.19A	0.16B	0.011	0.025	0.61	0.94
PC aa C42:5	0.30	0.25	0.02	0.30	0.25	0.02	0.045	0.88	0.94
PC ae C30:0	0.38B	0.42A	0.02	0.39B	0.43A	0.02	0.029	0.52	0.93
PC ae C32:1	1.4^b^	1.7^a^	0.10	1.4^b^	1.8^a^	0.10	0.003	0.76	0.76
PC ae C32:2	0.32	0.35	0.02	0.31	0.36	0.02	0.08	0.94	0.65
PC ae C34:0	0.85	0.96	0.06	0.83B	0.97A	0.06	0.043	0.88	0.68
PC ae C34:1	8.47^b^	11.9^a^	0.58	8.9	12.0	0.64	<0.001	0.39	0.41
PC ae C36:0	1.6B	2.0A	0.14	1.6B	2.0A	0.15	0.027	0.83	0.91
PC ae C36:1	7.6^b^	10.9^a^	0.69	6.5	10.3	0.70	<0.001	0.14	0.36
PC ae C36:2	4.9^b^	6.0^a^	0.38	4.5	6.0	0.39	0.004	0.43	0.46
PC ae C36:3	3.3^b^	3.9^a^	0.19	3.3^b^	3.9^a^	0.19	0.008	0.76	0.94
PC ae C36:5	1.8	1.6	0.09	1.7	1.6	0.09	0.06	0.89	0.94
PC ae C38:0	1.2B	1.4A	0.09	1.2^b^	1.5^a^	0.09	0.014	0.75	0.43
PC ae C38:1	1.4^b^	1.7^a^	0.11	1.4	1.6	0.11	0.026	0.68	0.57
PC ae C38:2	1.6^b^	2.1^a^	0.16	1.5	2.0	0.16	0.005	0.54	0.81
PC ae C38:3	2.5^b^	3.8^a^	0.22	2.4^b^	3.7^a^	0.22	<0.001	0.37	0.70
PC ae C42:1	0.60	0.67	0.04	0.57	0.63	0.05	0.09	0.31	0.88
PC ae C42:3	0.31	0.34	0.03	0.27	0.33	0.03	0.09	0.31	0.51
PC ae C44:5	0.38	0.33	0.03	0.39	0.33	0.03	0.046	0.85	0.99

^a^Values are least squares means ± SEM; control diet, n = 7; EMS diet, n = 6.

^b^C0, free carnitine; C2, acetylcarnitine; C14:1, tetradecenoylcarnitine; C16, hexadecanoylcarnitine; lysoPC a, lysophophatidylcholine with acyl residue C; SM C, sphingomyelin with C; SM(OH) C; PC aa C, phosphatidylcholine with diacyl residue sum C; PC ae C, phosphatidylcholine with acyl-alkyl residue sum C.

^c^Mean values within a row with different superscript letters were significantly different (*P*<0.05) per sampling time (fasting state or 60 min postprandially).

^d^Mean values within a row with different superscript capital letters tended to be different (*P*<0.1) per sampling time (fasting state or 60 min postprandially).

**Table 4:** Serum amino acids and biogenic amines (μmol/l) of pigs fed the enzymatically modified starch (EMS) diet or control diet in the fasting state and at 60 min postprandially.

	Fasting state			60 min postprandially			*P*-value		
Metabolite (μmol/l)^a^ ^–^ ^d^	Control diet	EMS diet	SEM	Control diet	EMS diet	SEM	Diet	Time	Diet × Time
Amino acids									
Alanine	395.4	426.0	33.27	775.3^b^	895.1^a^	34.41	0.049	<0.001	0.18
Arginine	179.9	174.3	8.38	232.6B	263.5A	8.70	0.23	<0.001	0.11
Asparagine	43.8	46.8	7.01	128.8^b^	150.7^a^	7.24	0.08	<0.001	0.154
Aspartate	19.6	21.4	2.37	25.9^b^	40.4^a^	2.44	0.007	<0.001	0.016
Citrulline	102.5	104.2	13.95	111.8^b^	164.3^a^	14.39	0.06	0.021	0.07
Glutamine	716.5	684.9	59.53	970.2	912.1	61.67	0.45	0.004	0.81
Glutamate	96.3	85.7	12.89	137.6	156.7	13.31	0.73	0.002	0.22
Glycine	1050.7	1264.7	78.06	1194.1	1119.2	81.05	0.45	0.99	0.15
Histidine	47.7	34.8	4.98	130.6	136.8	5.16	0.51	<0.001	0.09
Isoleucine	152.9	157.5	13.16	320.8B	360.4A	13.64	0.13	<0.001	0.20
Leucine	202.6	194.3	22.87	526.0B	595.6A	23.72	0.22	<0.001	0.13
Lysine	235.1	254.7	23.94	567.9	587.5	24.82	0.42	<0.001	1.00
Methionine	49.9	50.8	10.16	140.8	159.2	10.55	0.40	<0.001	0.44
Ornithine	76.3^b^	100.6^a^	5.55	148.2B	161.9A	5.72	0.007	<0.001	0.27
Phenylalanine	78.4	61.1	9.27	187.3B	213.9A	9.61	0.39	<0.001	0.08
Proline	284.7	273.9	29.60	682.6	727.2	30.75	0.61	<0.001	0.41
Serine	130.5	124.4	14.51	254.9B	294.3A	15.01	0.25	<0.001	0.13
Threonine	174.1	211.6	16.59	322.8^b^	391.4^a^	17.23	0.025	<0.001	0.40
Tryptophane	59.2	55.9	5.95	116.6	121.6	6.17	0.89	<0.001	0.50
Tyrosine	164.2	166.9	23.83	321.9^b^	373.6^a^	24.47	0.18	<0.001	0.21
Valine	357.8	370.9	24.80	567.2	614.7	25.67	0.23	<0.001	0.46
Biogenic amines									
ADMA	1.6	1.5	0.10	1.8B	1.8A	0.12	0.86	0.025	0.52
α-AAA	14.1	12.3	2.91	17.4	16.2	2.99	0.52	0.13	0.88
Carnosine	8.6	9.0	0.71	9.4	8.6	0.72	0.61	0.60	0.17
Creatinine	113.0	107.4	5.22	116.4	111.7	5.36	0.23	0.33	0.91
Histamine	0.80	0.74	0.26	1.6	1.4	0.27	0.57	0.020	0.76
Kynurenine	1.58	1.76	0.04	2.0	2.3	0.16	0.18	0.014	0.83
MetSO	1.00	0.76	0.33	6.0^a^	3.7^b^	0.35	0.020	<0.001	0.043
Putrescine	0.74^b^	0.92^a^	0.05	0.84	0.89	0.05	0.048	0.46	0.24
Sarcosine	15.1	14.4	1.55	15.6B	10.7A	1.61	0.10	0.29	0.18
SDMA	0.95	0.98	0.12	1.0	1.1	0.13	0.61	0.38	0.78
Serotonin	2.6	2.7	0.91	4.1	3.7	0.92	0.71	0.022	0.59
Spermidine	0.11	0.11	0.01	0.13	0.13	0.01	0.010	0.029	0.86
Taurine	41.4	39.4	5.03	43.3	45.3	5.18	0.18	0.35	0.62

^a^Values are least squares means ± SEM; control diet, n = 7; EMS diet, n = 6.

^b^ADMA, asymmetric dimethylarginine; α-AAA, α-amino adipate; DOPA, dihydroxyphenylalanine; Met-SO, methionine-sulfoxide; SDMA, symmetric dimethylarginine; TDMA, total dimethylarginine.

^c^Mean values within a row with different superscript letters were significantly different (*P*<0.05) per sampling time (fasting state or 60 min postprandially).

^d^Mean values within a row with different superscript capital letters tended to be different (*P*<0.1) per sampling time (fasting state or 60 min postprandially).

Of the detected metabolites, most intermediates of energy metabolism and acylcarnitines did not respond to EMS consumption but were raised (*P*<0.05) after meal consumption compared to the fasting state (Table 3). Only serum α-ketoglutarate concentration was higher in pigs fed EMS diet compared to pigs fed the control diet at 60 min postprandially (Table 3). Also, most amino acids and biogenic amines were elevated 60 min after feeding compared to fasting serum levels (Table 4). In contrast, sum of hexoses, sphingomyelins, almost all glycerophospholipids and lysophospholipids in serum were not affected by the feeding state (Table 3). Serum concentration of sum of hexoses tended (*P*<0.10) to be less in pigs fed the EMS diet compared to those fed the control diet at 60 min postprandially (Table 3), thereby being consistent with the MTT results for plasma glucose. The greatest effect of EMS was on serum profiles of phospholipids, including lysophosphatidylcholines, glycerophospholipids and sphingomyelins (Table 3) with effects going in the same direction in the fasting and postprandial state. There was a diet effect of EMS consumption on serum concentrations of certain long-chain sphingomyelins (SM(OH)C22:1, SM(OH)C24:1, SMC18:0, SMC18:1; SMC24:0, SMC24:1, SMC26:1) indicating that EMS diet lowered the concentrations of these sphingmyelins compared to the control diet. The effect of EMS on long-chain lysophosphatidylcholines C16:1, C17:0, and C18:1 was mostly an increase in serum concentrations, whereas lysophosphatidylcholines C18:2 and C20:3 concentration were decreased by EMS compared to the control diet. Serum concentrations of phosphatidylcholines with diacyl residues and acyl-alkyl residues were either increased or decreased by consumption of the EMS diet compared to the control diet with effects being similar in the fasting and postprandial state.

Of the targeted serum amino acids and biogenic amines, there was a general effect of diet on serum concentrations of alanine, aspartate, threonine, methionine-sulfoxide, spermidine and ornithine (*P*<0.05) and trends (*P*<0.10) for asparagine, citrulline, and sarcosine (Table 4). The effect of EMS consumption on amino acids and biogenic amines was more pronounced at 60 min postprandially than in the fasting state. Multiple comparison of data for each sampling time showed that concentrations of alanine, asparagine, aspartate, citrulline, threonine and tyrosine were greater (*P*<0.05) and those of arginine, isoleucine, leucine, ornithine, phenylalanine, serine, and asymmetric dimethylarginine tended to be raised (*P*<0.10) by EMS diet compared to the control diet at 60 min postprandially, whereas only serum ornithine and putrescine concentration was elevated (*P*<0.05) in pigs fed the EMS diet compared to pigs fed the control diet in the fasting state. In contrast, EMS diet decreased postprandial serum concentration of methionine-sulfoxide (*P*<0.05) and sarcosine (*P*<0.10) compared to the control diet. However, only for aspartate, citrulline (trend), histidine (trend), phenylalanine (trend) and methionine-sulfoxide, diet × time interactions were observed, indicating stronger effects of EMS in the postprandial phase compared to the fasting state.

## Discussion

To gain a deeper understanding of the modulating capacity of CMS on blood metabolites, we evaluated the effect of EMS on the fasting and early postprandial serum metabolome and postprandial dynamics of blood glucose, insulin and lipids in jugular vein-catheterized pigs. Our results support the theory of rapid modification of blood metabolite profiles in response to dietary alterations. Although our present blood metabolite concentrations did not mirror intestinal absorption [5], our data provide valuable information on the systemic response to EMS in comparison to the control starch in pigs. Also, we did not measure metabolite fluxes and can therefore only assume whether uptake or release of blood metabolites was changed by EMS ingestion. Most importantly, pigs fed the EMS diet showed clearly discernible changes in serum phospholipid and amino acid metabolome profiles in fasting and early postprandial state and postprandial serum lipid dynamics during MTT compared to serum profiles of pigs fed the control diet. These findings are in line with previously reported impacts of RS2 and RS4 on body fat metabolism in humans, pigs and rodents [6–8]. In contrast to that, present glucose and insulin dynamics did not comply with characteristic effects of RS intake of reduced serum glucose and insulin concentrations previously observed in humans, pigs and rodents in short- and long-term studies [3–7,24,25]. Indeed, the immediate insulin and glucose response was greater in pigs fed the EMS diet compared to pigs fed the control diet, although AUCs 0–480 min for insulin and glucose indicated similar responses for the whole MTT period for both diets. Therefore, the present findings support the concept that due to the specific modification of the starch molecule structure effects from one RS4 cannot be transferred to other CMS, rendering it necessary to evaluate physiological effects of CMS separately to describe potential RS properties [2,11]. In using a commercial enzymatic assay kit developed to determine naturally occurring RS types, it was not surprising that the amount of RS in the EMS diet that we could detect was very small. Overall, increasing the branching of the amylopectin fraction of the starch molecule in the EMS apparently did not yield the expected moderation in glucose release after ingestion compared to the control starch. From the chemical modification, EMS would have been less hydrolyzable for intestinal α-amylase. Besides the increased branching of the starch, the enzymatic treatment also reduced the overall molecular weight of EMS compared to the control starch. This might have accelerated the digestion of the rapidly digestible starch fraction of EMS, thereby leading to a faster glucose release and stronger insulin response in pigs fed the EMS diet within the first 60 min after feeding as compared to pigs fed the control diet. Possibly, sucrose-isomaltase activity at the brush border membrane could partly compensate for the reduced starch hydrolysis by α-amylase [26]. As the present catheterization model did not allow measuring net portal glucose absorption, our data do not let us distinguish whether the fluctuation in plasma glucose in pigs fed the EMS diet until about 210 min of the MTT was related to gastric emptying, first pass nutrition or insulin-mediated peripheral glucose uptake.

Intestinally absorbed glucose is proportionally converted to l-lactate in the portal-drained viscera during high glucose availability in the porcine gut [5]. Thus, the close relationship of plasma lactate to visceral glucose metabolism likely explains the similarity in the courses of plasma lactate and serum insulin during the MTT in pigs fed the control diet. Yet, this was not entirely true for pigs fed the EMS diet whose plasma lactate peaked twice in the second half of the MTT. In considering that less glucose was intestinally available with progressing time after feeding and the higher proportion of low-digestible starch in the EMS diet, increased intestinal lactate formation by microbial action on EMS might explain the elevated plasma lactate concentrations in the late hours of the MTT [2,5].

Similar to previous findings for RS2 in long-term studies [6,8,27], the EMS diet had a lowering effect on postprandial serum triglycerides and as a tendency also on cholesterol compared to the control diet. In contrast to previous studies [28], however, fasting triglycerides and cholesterol were not improved by the EMS diet compared to the control diet. The main underlying mechanism of RS to reduce blood cholesterol was thought to be increased intestinal viscosity and bile acid excretion due to RS intake [8,27,28]. Moreover, enhanced intestinal fermentation of RS and subsequent increased intestinal SCFA absorption and systemic metabolism has been also reported to contribute to fasting and postprandial blood triglycerides and cholesterol [25]. Intestinal fermentation parameters as well as serum SCFA were not measured in the present catheterized pigs; therefore, we can only speculate about a relation between intestinal fermentation and serum lipids. It may be added here that our unpublished data from a pig slaughter model and feeding growing pigs the experimental diets *ad libitum* showed that the EMS diet did not enhance intestinal fermentation compared to the control diet. It is noteworthy that the chemical modification of the starch structure may not only render the starch less digestible for the host enzymes, but it may also limit the starch hydrolysis by bacterial amylases [2].

There is evidence that the impact of RS intake on blood lipids is associated with changes in hepatic and peripheral fat metabolism, such as lipolysis and fatty acid oxidation, and lower levels of incretins, which has been linked to the RS-related reduction in serum insulin [6,7,27]. Indeed, serum insulin is the major player in postprandial metabolism of glucose, fat as well as of amino acids [29]. Insulin secretion after a meal enhances the rate of fatty acid synthesis and lipogenesis in hepatocytes and adipocytes via insulin-dependent up-regulation of transcription factor expression such as PPAR-γ and SREBP-1 [30,31]. However, we did not find lower insulin concentrations during the MTT. It should be mentioned here that we did not discriminate between the various lipoprotein fractions and can therefore not deduce whether EMS intake triggered mainly alterations in triglyceride synthesis, degradation or removal from the blood into hepatocytes or adipocytes when compared to the control starch. The present drop in serum NEFA and triglycerides in the first 60 min after feeding likely mirrored the insulin-mediated suppression of fat mobilization and lipolysis via suppression of hormone-sensitive lipase [31]. In addition, postprandial insulin secretion slows down mitochondrial fat oxidation by inhibiting carnitine palmitoyltransferase (CPT1) activity due to increased malonyl-CoA concentrations present during hyperglycemia [30]. Hence, the transfer of long-chain fatty acids into mitochondria via CPT1 is inhibited [30] which might have been indicated by the increased serum concentration of palmitoylcarnitine 60 min after feeding and caused by the increased insulin concentration in pigs fed the EMS diet compared to pigs fed the control diet. Likewise, elevated serum concentrations of free carnitine and acylcarnitines after feeding might hint at a reduced mitochondrial fatty acid transport in the early postprandial phase. Due to the severe impact of insulin on lipolysis, effects of RS on adipose tissue lipolysis and fat oxidation mostly become evident in the late postprandial period when insulin concentrations reach preprandial concentrations [6,32]. In this regard, elevated serum NEFA concentrations after the overnight fast and at 8 hours of the MTT may have indicated an enhanced fatty acid release in pigs fed the EMS diet compared to pigs fed the control diet.

An interesting and novel aspect of the present study was that long-chain lysophosphatidylcholines, glycerophospholipids and sphingolipids were not influenced by meal consumption, but were modified by EMS in a similar manner in the fasting state and at 60 min postprandially. These phospholipid groups are important structural components of plasma lipoproteins and cell membranes, being involved in the regulation of cell function, membrane protein trafficking and inflammation [33]. Lysophospholipids are derived from phosphatidylcholines during LDL oxidation, and saturated lysophospholipids were recognized to exert pro-inflammatory effects and impair insulin signaling [34,35]. In addition, alterations in blood phospholipid and lysophospholipid profiles are linked to obesity and insulin resistance [16,36–38]. To our knowledge, RS-related changes in phospholipid metabolome profiles were scarcely reported before. Our findings suggest a constant alteration in the synthesis or breakdown of sphingolipids and phospholipids in pigs fed the EMS diet when compared to pigs fed the control diet, independently of the feeding state. High insulin concentrations were reported to induce sphingomyelinase, down-regulate sphingomyelin synthase or induce phospholipase A_2_ and lecithin:cholesterol acyltransferase, leading to decreased plasma concentrations of sphingolipids, lysophosphatidylcholines and phospholipids [16]. Because in the present experiment the EMS effect on the phospholipid metabolome was not limited to the early postprandial state but was maintained during the fasting state, the present findings might not be simply explained via an increased serum insulin concentration in the immediate postprandial phase in pigs fed the EMS diet compared to those fed the control diet. Other intestinal and metabolic factors such as gut hormone profiles and intestinally absorbed SCFA may have contributed to the changes in phospholipid metabolome as well [16,25,39,40].

Especially postprandial levels of leucine together with insulin act as signals of increased amino acid and energy availability; thereby stimulating protein synthesis and simultaneously inhibiting proteolysis in muscle tissue [41–43]. Thus, elevated serum amino acids and certain biogenic amines (i.e., histamine, kynurenine, serotonin and spermidine) most likely originated from meal consumption at 60 min postprandially compared to the fasting state and were not due to increased muscle protein turnover. Additionally, the present dietary protein source (i.e., casein) was of high biological quality [20] and thus, absorbed amino acids should have been efficiently used for muscle protein synthesis. An interesting observation was that pigs fed the EMS diet had elevated serum concentrations of certain amino acids compared to pigs fed the control diet at 60 min postprandially. With high leucine and insulin concentrations preventing muscle proteolysis, the question arises whether EMS intake caused these elevated serum concentrations due to enhanced intestinal absorption or modification in muscle tissue uptake. It may be difficult to fully answer the underlying mode of action from the present data. Elevated serum concentrations of ornithine and putrescine in the fasting state and of arginine, aspartate, citrulline and ornithine 60 min postprandial with the EMS diet may hint at an increased amino acid deamination via the urea cycle, indicating a nutritional oversupply with amino acids as compared to the control diet. Evidence from rodents shows that an increase in luminal glucose concentrations down-regulates H^+^-coupled peptide transporter expression and dipeptide transport in the small intestine [44]. Thus, it might be feasible that differences in intestinal glucose release from the EMS diet compared to the control diet might have influenced peptide and amino acid absorption in the present study. By considering the great immediate insulin concentration, we would have rather expected a decrease in amino acid absorption with the EMS diet compared to the control diet. In this regard, additive effects of glucose and single amino acid (leucine and phenylalanine) intake on serum insulin concentration have been reported in humans [45]. Both leucine and phenylalanine were among the amino acids which concentrations were elevated by EMS diet, and might have theoretically added to the present immediate insulin response after the meal.

In conclusion, our results indicate a potential for EMS to attenuate postprandial raise in serum lipids. More importantly, our data suggest a constant alteration in the synthesis or breakdown of sphingolipids and phospholipids in pigs in response to EMS consumption, independently of the feeding state. However, the decrease in serum insulin and glucose commonly observed with RS ingestion and which was previously linked to the lowering of serum lipids was not found in pigs fed the EMS diet compared to pigs fed the control diet. Thus, more research is necessary to disclose possible underlying mechanisms responsible for observed changes in serum lipid profiles and whether there were interactions between serum insulin, lipid and amino acid profiles, and intestinal fermentation and incretin secretion. Also, results need to be confirmed for lower dietary inclusion levels of EMS. Finally, results supported the rapidity by which porcine blood metabolite profiles adapt to shifts in dietary starch source.

## Supporting Information

S1 Table Serum concentrations (μmol/l) of lysophosphatidylcholines and sphingomyelins in pigs fed the enzymatically modified starch (EMS) diet or control diet in the fasting state and at 60 min postprandially. (DOCX)

S2 Table Serum phosphatidylcholine concentrations (μmol/l) in pigs fed the enzymatically modified starch (EMS) diet or control diet in the fasting state and at 60 min postprandially. (DOCX)
