# Supplementary material for: Enzymatically Modified Starch Ameliorates Postprandial Serum Triglycerides and Lipid Metabolome in Growing Pigs
Source: PLoS One. 2015 Jun 15;10(6):e0130553. doi: 10.1371/journal.pone.0130553 (PMC4468079; doi:10.1371/journal.pone.0130553)
Supplement: S1 Table — (DOCX) [file pone.0130553.s001.docx]

**Supporting Information - Metzler-Zebeli et al.**

**S1 Table. Serum concentrations (µmol/l) of lysophosphatidylcholines and sphingomyelins in pigs fed the enzymatically modified starch (EMS) diet or control diet in the fasting state and at 60 min postprandially.**

|  | Fasting | | |  | 60 min postprandially | | |  | *P*-value | | |
| --- | --- | --- | --- | --- | --- | --- | --- | --- | --- | --- | --- |
| Item (µmol/l)^a-d^ | Control | EMS | SEM |  | Control | EMS | SEM |  | Diet | Time | Diet × Time |
| Lysophospholipids |  |  |  |  |  |  |  |  |  |  |  |
| lysoPC a C16:0 | 27.4 | 28.3 | 0.99 |  | 26.8 | 26.9 | 1.02 |  | 0.64 | 0.32 | 0.67 |
| lysoPC a C16:1 | 2.0^b^ | 3.8^a^ | 0.31 |  | 1.8^b^ | 4.0^a^ | 0.32 |  | <0.001 | 0.89 | 0.36 |
| lysoPC a C17:0 | 0.60^b^ | 0.72^a^ | 0.04 |  | 0.59 | 0.71 | 0.04 |  | 0.012 | 0.78 | 0.98 |
| lysoPC a C18:0 | 14.9 | 14.3 | 0.69 |  | 15.2 | 14.4 | 0.72 |  | 0.29 | 0.76 | 0.87 |
| lysoPC a C18:1 | 21.8^b^ | 30.1^a^ | 1.67 |  | 19.3 | 31.0 | 1.77 |  | <0.001 | 0.54 | 0.20 |
| lysoPC a C18:2 | 5.5^A^ | 4.7^B^ | 0.30 |  | 5.7^a^ | 4.6^b^ | 0.31 |  | 0.015 | 0.75 | 0.52 |
| lysoPC a C20:3 | 2.1^b^ | 2.6^a^ | 0.13 |  | 2.1 | 2.6 | 0.15 |  | 0.005 | 0.36 | 0.68 |
| lysoPC a C20:4 | 5.7 | 5.5 | 0.30 |  | 5.4 | 5.2 | 0.25 |  | 0.51 | 0.21 | 0.88 |
| lysoPC a C26:0 | 0.15 | 0.15 | 0.01 |  | 0.14 | 0.14 | 0.01 |  | 0.84 | 0.78 | 1.00 |
| lysoPC a C26:1 | 0.08 | 0.09 | 0.01 |  | 0.09 | 0.10 | 0.01 |  | 0.36 | 0.47 | 0.88 |
| lysoPC a C28:0 | 0.19 | 0.18 | 0.01 |  | 0.19 | 0.20 | 0.01 |  | 1.00 | 0.46 | 0.71 |
| lysoPC a C28:1 | 0.13 | 0.14 | 0.01 |  | 0.14 | 0.15 | 0.01 |  | 0.65 | 0.37 | 0.81 |
|  |  |  |  |  |  |  |  |  |  |  |  |
| Sphingomyelins |  |  |  |  |  |  |  |  |  |  |  |
| SM(OH)C14:1 | 1.3 | 1.3 | 0.09 |  | 1.2 | 1.2 | 0.09 |  | 1.00 | 0.58 | 0.92 |
| SM(OH)C16:1 | 1.7 | 1.7 | 0.11 |  | 1.7 | 1.7 | 0.12 |  | 0.77 | 0.86 | 0.73 |
| SM(OH)C22:1 | 3.0 | 2.7 | 0.18 |  | 2.9 | 2.5 | 0.19 |  | 0.09 | 0.36 | 0.75 |
| SM(OH)C22:2 | 2.1 | 2.2 | 0.13 |  | 2.1 | 2.0 | 0.14 |  | 0.92 | 0.60 | 0.46 |
| SM(OH)C24:1 | 0.38^A^ | 0.29^B^ | 0.03 |  | 0.38^a^ | 0.26^b^ | 0.03 |  | 0.015 | 0.62 | 0.59 |
| SMC16:0 | 59.2 | 57.2 | 3.14 |  | 59.3 | 54.6 | 3.12 |  | 0.26 | 0.66 | 0.62 |
| SMC16:1 | 5.6 | 5.8 | 0.40 |  | 5.7 | 5.4 | 0.42 |  | 0.86 | 0.63 | 0.47 |
| SMC18:0 | 14.8^A^ | 12.9^B^ | 0.97 |  | 14.5 | 12.7 | 0.99 |  | 0.032 | 0.68 | 0.98 |
| SMC18:1 | 4.2 | 3.9 | 0.25 |  | 4.3 | 3.9 | 0.26 |  | 0.08 | 0.90 | 0.83 |
| SMC20:2 | 0.14 | 0.14 | 0.01 |  | 0.14 | 0.13 | 0.01 |  | 0.81 | 0.80 | 0.56 |
| SMC24:0 | 12.3 | 10.9 | 0.75 |  | 12.5 | 10.1 | 0.77 |  | 0.031 | 0.63 | 0.48 |
| SMC24:1 | 27.8^A^ | 24.0^B^ | 1.52 |  | 27.5 | 22.6 | 1.57 |  | 0.020 | 0.52 | 0.67 |
| SMC26:1 | 0.11^a^ | 0.03^b^ | 0.02 |  | 0.06 | 0.04 | 0.02 |  | 0.05 | 0.47 | 0.24 |

^a^Values are least squares means ± SEM; control diet, n=7; EMS diet, n=6.

^b^lysoPC a, lysophophatidylcholine with acyl residue C; SM C, sphingomyelin with C; SM(OH) C.

^c^Mean values within a row with different superscript letters were significantly different (*P*<0.05) per sampling time (fasting state or 60 min postprandially).

^d^Mean values within a row with different superscript capital letters tended to be different (*P*<0.1) per sampling time (fasting state or 60 min postprandially).
