# Supplementary material for: Enzymatically Modified Starch Ameliorates Postprandial Serum Triglycerides and Lipid Metabolome in Growing Pigs
Source: PLoS One. 2015 Jun 15;10(6):e0130553. doi: 10.1371/journal.pone.0130553 (PMC4468079; doi:10.1371/journal.pone.0130553)
Supplement: S2 Table — (DOCX) [file pone.0130553.s002.docx]

**Supporting Information - Metzler-Zebeli et al.**

**S2 Table. Serum phosphatidylcholine concentrations (µmol/l) in pigs fed the enzymatically modified starch (EMS) diet or control diet in the fasting state and at 60 min postprandially.**

|  | Fasting | | |  | 60 min postprandially | | |  | *P*-value | | |
| --- | --- | --- | --- | --- | --- | --- | --- | --- | --- | --- | --- |
| Item (µmol/l)^a-d^ | Control | EMS | SEM |  | Control | EMS | SEM |  | Diet | Time | Diet × Time |
| PC aa C24:0 | 0.09 | 0.10 | 0.01 |  | 0.09 | 0.09 | 0.01 |  | 0.50 | 0.25 | 0.33 |
| PC aa C28:1 | 0.06 | 0.06 | 0.03 |  | 0.59 | 0.59 | 0.03 |  | 0.90 | 0.52 | 0.97 |
| PC aa C30:0 | 1.5^b^ | 1.8^a^ | 0.12 |  | 1.4^b^ | 1.7^a^ | 0.12 |  | 0.010 | 0.31 | 0.89 |
| PC aa C32:0 | 6.8 | 7.1 | 0.38 |  | 6.5 | 6.7 | 0.39 |  | 0.40 | 0.26 | 0.93 |
| PC aa C32:1 | 10.6 | 19.5 | 1.66 |  | 8.3 | 18.1 | 1.70 |  | <0.001 | 0.12 | 0.68 |
| PC aa C32:2 | 0.30^b^ | 0.72^a^ | 0.10 |  | 0.14 | 0.67 | 0.10 |  | 0.001 | 0.19 | 0.50 |
| PC aa C32:3 | 0.09 | 0.10 | 0.01 |  | 0.82 | 0.09 | 0.01 |  | 0.27 | 0.29 | 0.75 |
| PC aa C34:1 | 218.8^b^ | 269.3^a^ | 13.59 |  | 200.1^b^ | 255.3^a^ | 14.01 |  | 0.005 | 0.18 | 0.83 |
| PC aa C34:2 | 71.3 | 63.0 | 4.01 |  | 70.2^a^ | 58.2^b^ | 4.12 |  | 0.021 | 0.36 | 0.57 |
| PC aa C34:3 | 3.4^b^ | 4.2^a^ | 0.31 |  | 3.0^b^ | 4.3^a^ | 0.32 |  | 0.009 | 0.54 | 0.42 |
| PC aa C34:4 | 0.27^b^ | 0.36^a^ | 0.03 |  | 0.22^b^ | 0.35^a^ | 0.03 |  | 0.007 | 0.29 | 0.53 |
| PC aa C36:1 | 126.5^b^ | 165.5^a^ | 9.68 |  | 110.9^b^ | 160.9^a^ | 9.99 |  | 0.003 | 0.25 | 0.52 |
| PC aa C36:2 | 101.4 | 98.2 | 7.01 |  | 94.8 | 94.8 | 7.24 |  | 0.80 | 0.42 | 0.80 |
| PC aa C36:3 | 41.5 | 44.9 | 2.38 |  | 39.2 | 42.2 | 2.46 |  | 0.16 | 0.25 | 0.93 |
| PC aa C36:4 | 71.2 | 69.1 | 4.08 |  | 65.4 | 63.5 | 4.20 |  | 0.54 | 0.10 | 0.97 |
| PC aa C36:5 | 4.1^B^ | 5.6^A^ | 0.50 |  | 3.3^b^ | 5.7^a^ | 0.52 |  | 0.008 | 0.46 | 0.35 |
| PC aa C36:6 | 0.15 | 0.19 | 0.02 |  | 0.11^b^ | 0.21^a^ | 0.02 |  | 0.011 | 0.75 | 0.18 |
| PC aa C38:0 | 45.2 | 44.2 | 3.03 |  | 43.5 | 41.8 | 3.14 |  | 0.65 | 0.48 | 0.91 |
| PC aa C38:4 | 128.6^A^ | 108.3^B^ | 7.87 |  | 121.5^A^ | 101.9^B^ | 8.13 |  | 0.028 | 0.33 | 0.96 |
| PC aa C38:5 | 40.7 | 40.6 | 2.62 |  | 36.7 | 39.2 | 2.72 |  | 0.65 | 0.29 | 0.60 |
| PC aa C38:6 | 20.5^A^ | 17.1^B^ | 1.36 |  | 18.5 | 16.1 | 1.39 |  | 0.029 | 0.17 | 0.74 |
| PC aa C40:2 | 0.72 | 0.79 | 0.07 |  | 0.76 | 0.69 | 0.07 |  | 0.99 | 0.63 | 0.35 |
| PC aa C40:3 | 1.5 | 1.3 | 0.12 |  | 1.5 | 1.8 | 0.12 |  | 0.044 | 0.76 | 0.31 |
| PC aa C40:4 | 11.7 | 12.2 | 1.0 |  | 11.9 | 10.99 | 1.04 |  | 0.83 | 0.58 | 0.43 |
| PC aa C40:5 | 22.5 | 19.9 | 1.38 |  | 21.1 | 18.5 | 1.43 |  | 0.09 | 0.28 | 0.93 |
| PC aa C40:6 | 18.8 | 16.1 | 1.13 |  | 16.42 | 14.9 | 1.17 |  | 0.08 | 0.11 | 0.57 |
| PC aa C42:0 | 0.12 | 0.11 | 0.01 |  | 0.12 | 0.11 | 0.01 |  | 0.14 | 0.83 | 0.85 |
| PC aa C42:1 | 0.11 | 0.10 | 0.01 |  | 0.12 | 0.12 | 0.01 |  | 0.99 | 0.049 | 0.24 |
| PC aa C42:2 | 0.15 | 0.13 | 0.01 |  | 0.15 | 0.13 | 0.01 |  | 0.05 | 0.83 | 0.68 |
| PC aa C42:4 | 0.18^A^ | 0.15^B^ | 0.01 |  | 0.19^A^ | 0.16^B^ | 0.01 |  | 0.025 | 0.61 | 0.94 |
| PC aa C42:5 | 0.30 | 0.25 | 0.02 |  | 0.30 | 0.25 | 0.02 |  | 0.045 | 0.88 | 0.94 |
| PC aa C42:6 | 0.29 | 0.27 | 0.02 |  | 0.29 | 0.25 | 0.02 |  | 0.13 | 0.63 | 0.63 |
| PC ae C30:0 | 0.38^B^ | 0.42^A^ | 0.02 |  | 0.39^B^ | 0.43^A^ | 0.02 |  | 0.029 | 0.52 | 0.93 |
| PC ae C30:2 | 0.073 | 0.071 | 0.004 |  | 0.076 | 0.074 | 0.004 |  | 0.66 | 0.42 | 0.91 |
| PC ae C32:1 | 1.4^b^ | 1.7^a^ | 0.10 |  | 1.4^b^ | 1.8^a^ | 0.10 |  | 0.003 | 0.76 | 0.76 |
| PC ae C32:2 | 0.32 | 0.35 | 0.02 |  | 0.31 | 0.36 | 0.022 |  | 0.08 | 0.94 | 0.65 |
| PC ae C34:0 | 0.85 | 0.96 | 0.06 |  | 0.83^B^ | 0.97^A^ | 0.062 |  | 0.043 | 0.88 | 0.68 |
| PC ae C34:1 | 8.5^b^ | 12.0^a^ | 0.58 |  | 8.9 | 12.0 | 0.64 |  | <0.001 | 0.39 | 0.41 |
| PC ae C34:2 | 3.8 | 3.9 | 0.18 |  | 3.8 | 4.0 | 0.19 |  | 0.44 | 0.49 | 0.61 |
| PC ae C36:0 | 1.6^B^ | 2.0^A^ | 0.14 |  | 1.6^B^ | 2.0^A^ | 0.15 |  | 0.027 | 0.83 | 0.91 |
| PC ae C36:1 | 7.6^b^ | 10.9^a^ | 0.69 |  | 6.5 | 10.3 | 0.70 |  | <0.001 | 0.14 | 0.36 |
| PC ae C36:2 | 4.9^b^ | 6.0^a^ | 0.38 |  | 4.5 | 6.0 | 0.39 |  | 0.004 | 0.43 | 0.46 |
| PC ae C36:3 | 3.3^b^ | 3.9^a^ | 0.19 |  | 3.3^b^ | 3.9^a^ | 0.19 |  | 0.008 | 0.76 | 0.94 |
| PC ae C36:4 | 4.4 | 4.3 | 0.18 |  | 4.4 | 4.3 | 0.18 |  | 0.61 | 0.96 | 0.81 |
| PC ae C36:5 | 1.8 | 1.6 | 0.09 |  | 1.7 | 1.6 | 0.09 |  | 0.06 | 0.89 | 0.94 |
| PC ae C38:0 | 1.2^B^ | 1.4^A^ | 0.09 |  | 1.2^b^ | 1.5^a^ | 0.09 |  | 0.014 | 0.75 | 0.43 |
| PC ae C38:1 | 1.4^b^ | 1.7^a^ | 0.11 |  | 1.4 | 1.6 | 0.11 |  | 0.026 | 0.68 | 0.57 |
| PC ae C38:2 | 1.6^b^ | 2.1^a^ | 0.16 |  | 1.5 | 2.0 | 0.16 |  | 0.005 | 0.54 | 0.81 |
| PC ae C38:3 | 2.5^b^ | 3.8^a^ | 0.22 |  | 2.4^b^ | 3.7^a^ | 0.22 |  | <0.001 | 0.37 | 0.70 |
| PC ae C38:4 | 9.6 | 10.1 | 0.53 |  | 9.1 | 9.8 | 0.54 |  | 0.18 | 0.29 | 0.86 |
| PC ae C38:5 | 6.0 | 5.7 | 0.30 |  | 5.7 | 5.7 | 0.31 |  | 0.50 | 0.61 | 0.50 |
| PC ae C38:6 | 1.5 | 1.6 | 0.08 |  | 1.4 | 1.5 | 0.09 |  | 0.10 | 0.29 | 0.52 |
| PC ae C40:0 | 4.9 | 5.4 | 0.36 |  | 4.4 | 5.0 | 0.37 |  | 0.13 | 0.21 | 0.83 |
| PC ae C40:1 | 1.2 | 1.2 | 1.30 |  | 1.2 | 1.2 | 1.32 |  | 0.26 | 0.11 | 0.26 |
| PC ae C40:2 | 0.58 | 0.60 | 0.04 |  | 0.56 | 0.57 | 0.04 |  | 0.65 | 0.43 | 0.81 |
| PC ae C40:3 | 0.92 | 0.89 | 0.06 |  | 0.88 | 0.83 | 0.07 |  | 0.57 | 0.44 | 0.83 |
| PC ae C40:4 | 2.6 | 2.5 | 0.16 |  | 2.5 | 2.4 | 0.17 |  | 0.85 | 0.47 | 0.95 |
| PC ae C40:5 | 2.5 | 2.5 | 0.15 |  | 2.4 | 2.5 | 0.15 |  | 0.88 | 0.44 | 0.88 |
| PC ae C40:6 | 1.6 | 1.7 | 0.09 |  | 1.5 | 1.6 | 0.10 |  | 0.17 | 0.23 | 0.36 |
| PC ae C42:1 | 0.60 | 0.67 | 0.04 |  | 0.57 | 0.63 | 0.05 |  | 0.089 | 0.31 | 0.88 |
| PC ae C42:2 | 0.55 | 0.59 | 0.05 |  | 0.47 | 0.55 | 0.05 |  | 0.16 | 0.14 | 0.65 |
| PC ae C42:3 | 0.31 | 0.34 | 0.03 |  | 0.27 | 0.33 | 0.03 |  | 0.099 | 0.31 | 0.51 |
| PC ae C42:4 | 0.29 | 0.28 | 0.02 |  | 0.28 | 0.24 | 0.02 |  | 0.17 | 0.27 | 0.43 |
| PC ae C44:3 | 0.12 | 0.12 | 0.01 |  | 0.11 | 0.11 | 0.09 |  | 0.88 | 0.35 | 1.00 |
| PC ae C44:4 | 0.13 | 0.11 | 0.01 |  | 0.11 | 0.12 | 0.01 |  | 0.76 | 0.79 | 0.16 |
| PC ae C44:5 | 0.38 | 0.33 | 0.03 |  | 0.39 | 0.33 | 0.03 |  | 0.046 | 0.85 | 0.99 |
| PC ae C44:6 | 0.17 | 0.16 | 0.01 |  | 0.16 | 0.16 | 0.01 |  | 0.42 | 0.58 | 0.68 |

^a^Values are least squares means ± SEM; control diet, n=7; EMS diet, n=6.

^b^PC aa C, phosphatidylcholine with diacyl residue sum C; PC ae C, phosphatidylcholine with acyl-alkyl residue sum C.

^c^Mean values within a row with different superscript letters were significantly different (*P*<0.05) per sampling time (fasting state or 60 min postprandially).

^d^Mean values within a row with different superscript capital letters tended to be different (*P*<0.1) per sampling time (fasting state or 60 min postprandially).
